# Myocarditis in SARS-CoV-2: A Meta-Analysis

**DOI:** 10.7759/cureus.48059

**Published:** 2023-10-31

**Authors:** Ranel Thaker, James Faraci, Sierra Derti, John F Schiavone

**Affiliations:** 1 Internal Medicine, Lake Erie College of Osteopathic Medicine, Elmira, USA; 2 Surgery, Lake Erie College of Osteopathic Medicine, Elmira, USA

**Keywords:** covid-19 and myocarditis, vaccine-associated myocarditis, sars-cov-2, covid-19 vaccine, covid-19

## Abstract

There has been a rise in cardiovascular events following the onset of the severe acute respiratory syndrome coronavirus 2 (SARS-CoV-2) infection, a strain that caused coronavirus disease 2019 (COVID-19). Although rare, there has been an increase in reports of myocarditis secondary to both individuals infected by the strain and those who received the COVID-19 mRNA vaccine. The focus of this study is to determine the risk of myocarditis associated with the COVID-19 vaccine and SARS-CoV-2 infection.

Relevant literature was collected using the search engines PubMed, Google Scholar, and the WHO Global Literature on Coronavirus Disease. Randomized controlled clinical trials and cohort studies reporting the risk of myocarditis induced by SARS-CoV-2 infection and COVID-19 vaccines were used. A meta-analysis was conducted using the inverse variance method using RevMan application software.

A meta-analysis of the compiled data showed a mean risk ratio of 4.74 (95% confidence interval (CI) = 2.40 to 9.36; p < 0.0000100), which indicates there is a significant difference in the risk of COVID-19-induced myocarditis in those with unspecified vaccination status compared to the non-infected population. A meta-analysis of the selected data found a mean risk ratio of 5.01 (95% CI = 4.14 to 6.08; p < 0.0000100), indicating a significant difference in the risk of COVID-19-induced myocarditis between those who are unvaccinated and the non-infected population. Upon a meta-analysis of the selected data set, a mean risk ratio of 2.55 (95% CI = 0.840 to 7.74; p = 0.100) was found, indicating no significant difference in the risk of vaccine-induced myocarditis between those with a vaccinated vaccination status and that of the non-infected population.

The result of this meta-analysis showed that infection with SARS-CoV-2 in unvaccinated patients carries a statistically significant increased risk of acquiring myocarditis while those receiving the vaccination do not share this same risk.

## Introduction and background

The severe acute respiratory syndrome coronavirus 2 (SARS-CoV-2) is a novel coronavirus that caused a worldwide pandemic with over 6.8 million related deaths by the end of 2020 [1]. Coronavirus disease 2019 (COVID-19) produces a pneumonia-like illness with symptoms ranging from mild to severe and possibly even fatal. Serious respiratory outcomes are associated with comorbidities such as chronic obstructive pulmonary disease, diabetes mellitus, and heart failure. COVID-19 vaccination was developed in the hope of protecting millions of individuals from the new onset effects of this virus. These vaccines sought to develop mRNA spike proteins that could be translated in the cytoplasm, and, in turn, create a large number of antibodies to be able to neutralize or destroy the virus.

Myocarditis is a relatively rare inflammatory disease affecting the myocardium caused by a variety of etiologic agents, including viruses, toxins, medications, and other inflammatory processes, all of which affect the myocardium, leading to heart failure and sudden death in a subgroup of patients. Typically, myocarditis follows viral infections most often caused by enteroviruses, specifically Coxsackie group B, and, less commonly, adenoviruses, parvovirus B19, hepatitis C, cytomegalovirus, and human immunodeficiency virus. The viruses are thought to infiltrate the myocardium and produce an immunologic activation within the cells causing either cytopathic effect or direct immune damage. It was estimated that about nine cases per 100,000 patients developed myocarditis without COVID-19 and about 150 per 100,000 patients were seen to develop myocarditis when infected with COVID-19. Myocarditis in 2020 was seen during inpatient encounters 42% more frequently following the start of the COVID-19 pandemic than it was in 2019. It was estimated that patients with COVID-19 from March 2020 through January 2021 had, on average, 15.7 times greater risk of developing myocarditis compared to those without COVID-19 [2]. Myocarditis was a relatively rare complication before the increase in numbers seen following the onset of the COVID-19 pandemic. Although rare in the non-COVID-19 population, the risk of post-COVID-19 myocarditis has been a growing concern with an increased incidence of events in both the unvaccinated and vaccinated populations.

It was recently proposed that unvaccinated patients, regardless of gender, exhibited a higher risk of myocarditis. Interestingly, males aged 12-17 years were found to have a higher risk of developing myocarditis with COVID-19. Those who received their second dose of vaccine were also seen to be at an increased risk. Additionally, the risk of myocarditis in unvaccinated 12-17-year-old males was found to be 1.8-5.6 times higher [3]. Other sources suggest that there was a seven times increased risk of developing myocarditis in the unvaccinated population versus the vaccinated population with men, once again, being at higher risk [4]. An additional study investigated COVID-19-negative patients who received the vaccine and postulated that there may be an increased risk of myocarditis in vaccinated patients aged 18 or older. In one study, of the patients who were vaccinated with one or two doses, 15 resulted in hospital admissions for myocarditis with no previous cardiac history. All patients had an unremarkable hospital stay and did not require readmission [5]. Of note, younger males were believed to warrant further investigation. Seemingly, myocarditis can be a complication of both COVID-19 and the mRNA vaccine.

Given the recent hesitancy and concern about receiving the COVID-19 vaccine secondary to possible adverse events, such as myocarditis, we set out to investigate the risk of vaccine-induced myocarditis and asses the vaccine’s mitigating effects on acquired myocarditis related to the COVID-19 infection.

## Review

Methodology

**Figure 1:**
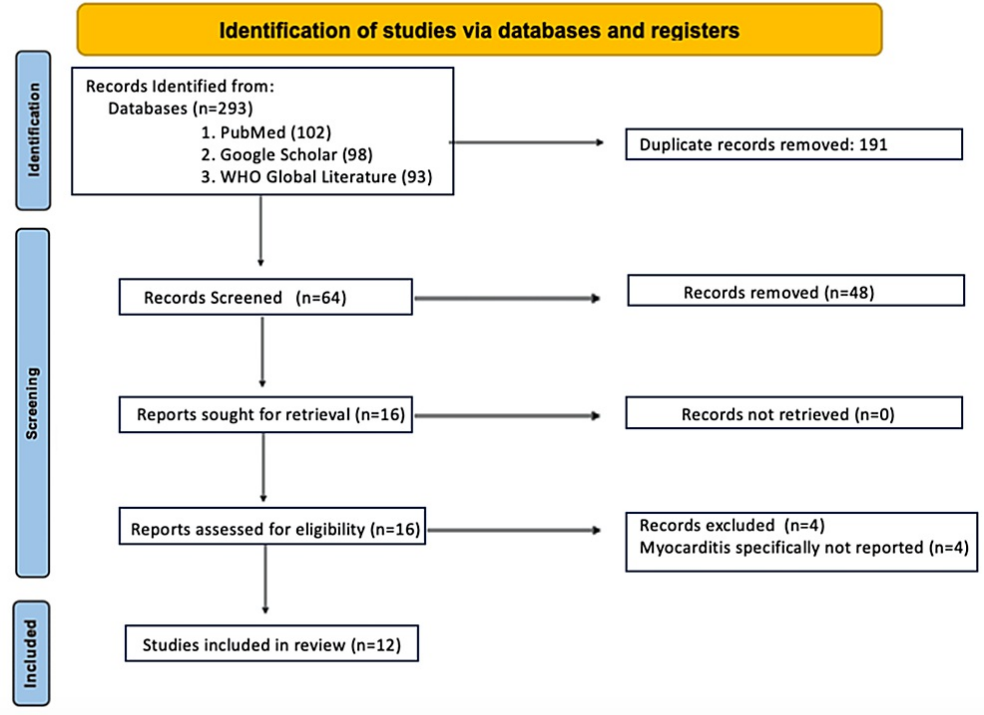
Preferred Reporting Items for Systematic Reviews and Meta-Analyses flowchart for study selection.

We included randomized controlled clinical trials and cohort studies that reported the risk of myocarditis induced by the SARS-CoV-2 infection and COVID-19 vaccines. We performed an electronic search on PubMed, Google Scholar, and the WHO Global Literature on Coronavirus Disease to identify relevant articles. The following keyword search terms were used: [“myocarditis” or cardiac disease] AND [“COVID-19” OR “SARS-CoV-2” OR “Coronavirus”]; [“myocarditis” or “cardiac disease”] AND [“COVID-19 Vaccination” OR “SARS-CoV-2 Vaccination OR “Coronavirus Vaccine”]. There were no exclusions based on the size or type of study. There were also no exclusions based on sex, age, or race. The study selection process is illustrated in Figure 1. A total of 293 studies were screened, and 12 studies were included in the review, as seen in Figure 1. The characteristics of these studies are presented in Table 1. The primary outcome was COVID-19-induced myocarditis in patients with an unknown vaccination status. The secondary outcome was COVID-19-induced myocarditis in unvaccinated patients. The tertiary outcome was COVID-19 vaccine-induced myocarditis. The distribution was normalized using standard errors calculated using the following equation: standard error = (upper limit - lower limit)/3.92. A meta-analysis was conducted using the inverse variance method using RevMan application software.

**Table 1:** Characteristics of included studies.

References	Year of publication	Country	Risk of bias	Sample size	Patients with myocarditis	Cohort	Type of vaccine
Patone et al. [6]	2021	UK	Low	38,615,491	1,615	Vaccine	Pfizer/Moderna
Barda et al. [7]	2021	Israel	Low	884,828	21	Vaccine	Pfizer
Simone et al. [5]	2021	US	Low	2,392,924	15	Vaccine	Pfizer/Moderna
Couchana et al. [8]	2021	US/Europe, Latin America, Africa	Low	2,277	1,241	Vaccine	Pfizer/Moderna
Le Vu et al. [9]	2022	France	Low	421	51	Vaccine	Pfizer
Won Lee et al. [10]	2022	US	Low	162,993	263	Vaccine	Pfizer
Xie et al. [11]	2022	US	Low	153,760	27,003	COVID-19 infection in unvaccinated patients	N/A
Barda et al. [7]	2021	Israel	Low	233,392	93,812	COVID-19 infection in unvaccinated patients	N/A
Boehmer et al. [2]	2021	US	Low	1,452,773	5,069	COVID-19 infection in unvaccinated patients	N/A
Wang et al. [12]	2022	US	Low	123	95	COVID-19 infection in unvaccinated patients	N/A
Patone et al. [6]	2021	UK	Low	2,315,669	N/A	COVID-19 infection, unspecified vaccination status	N/A
Murk et al. [13]	2020	US	Low	70,288	Unknown	COVID-19 infection, unspecified vaccination status	N/A
Daniels et al. [14]	2021	US	Low	2,810	37	COVID-19 infection, unspecified vaccination status	N/A
Cohen et al. [15]	2022	US	Low	27,698	N/A	COVID-19 infection, unspecified vaccination status	N/A
Xie et al. [11]	2022	US	Low	153,760	N/A	COVID-19 infection, unspecified vaccination status	N/A

Results

COVID-19-Induced Myocarditis in Patients With an Unspecified Vaccination Status

**Figure 2:**
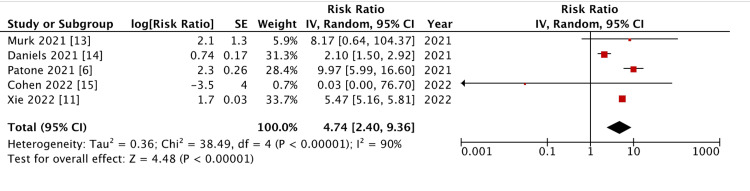
Forest plot on COVID-19-induced myocarditis. The vaccination status of patients is unspecified.

All studies were included in this review based on the previously determined exclusion criteria. As there was significant heterogeneity (p < 0.0000100, I^2^ = 90%), a reverse variance model was utilized for analysis. This heterogeneity may be a consequence of the smaller sample size of the meta-analysis, and therefore, was still considered for analysis. A meta-analysis of the compiled data showed a mean risk ratio of 4.74 (95% confidence interval (CI) = 2.40 to 9.36; p < 0.0000100), indicating a significant difference in the relative risk of COVID-19-induced myocarditis in those with an unspecified vaccination status compared to the non-infected population (Figure 2).

COVID-19-Induced Myocarditis in Patients With an Unvaccinated Status

**Figure 3:**
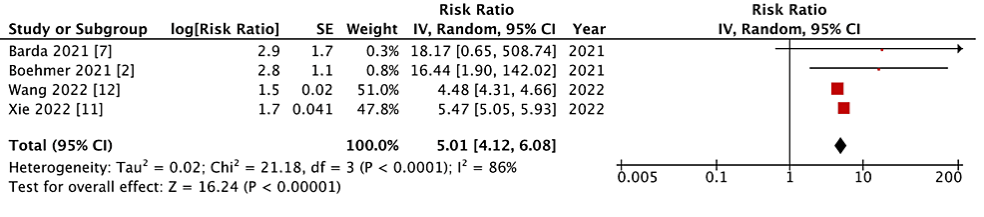
Forest plot on COVID-19-induced myocarditis in unvaccinated patients.

No studies were excluded from this meta-analysis based on the exclusion criteria. As there was significant heterogeneity (p = <0.000100, I^2^ = 86%), a reverse variance model was used for analysis. A meta-analysis of the selected data found a mean risk ratio of 5.01 (95% CI = 4.14 to 6.08; p < 0.0000100). This indicates a significant difference in the risk of COVID-19-induced myocarditis between those who are unvaccinated and the non-infected population (Figure 3).

COVID-19-Induced Myocarditis in Patients With a Vaccinated Status

**Figure 4:**
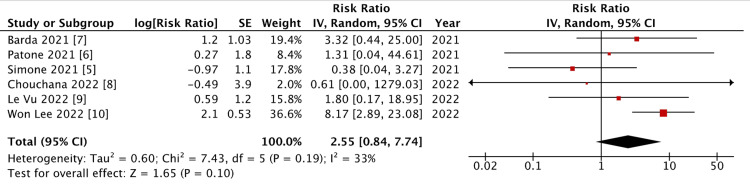
Forest plot on COVID-19 vaccine-associated myocarditis.

All studies identified upon review of the literature were included based on the determined exclusion criteria. Although there was some degree of non-significant heterogeneity (p = 0.190, I^2^ = 33%), a reverse variance model was still utilized. Upon a meta-analysis of the selected data set, a mean risk ratio of 2.55 (95% CI = 0.840 to 7.74; p = 0.100) was found, indicating no significant difference in the risk of COVID-19-induced myocarditis between those who are vaccinated and the non-infected population (Figure 4).

Discussion

In this meta-analysis, we found a significant increase in the relative risk of suffering from SARS-CoV-2-induced myocarditis when there was no confirmation of vaccination (Figures 2, 3). This is contrary to the finding that there is no significant increase in risk of this sequelae among those with a confirmed history of COVID-19 vaccination (Figure 4). This further supports previous primary analyses that found the risk of myocarditis associated with exposure to COVID-19 through infection is far greater than that associated with vaccination [12]. A 2022 review article concluded that the incidence of SARS-CoV-2-induced myocarditis is estimated to be approximately 100 times greater than that of COVID-19 vaccine-induced myocarditis [16]. This supports the findings of this analysis and speaks to the subsequent conclusions drawn.

It is important to juxtapose the results of this meta-analysis with previous studies that have provided results that directly oppose the conclusions shown here. There have been multiple studies that found no significant difference in the rates or risk of SARS-CoV-2-induced myocarditis in individuals without vaccination and the non-infected population. One study concluded that many of the cases of SARS-CoV-2-induced myocarditis may be due to Takotsubo cardiomyopathy, which is clinically indistinguishable from SARS-CoV-2-induced myocarditis. Therefore, it was concluded that one cannot be exclusively assigned to a unifactorial cause [17]. However, the increased prevalence of myocarditis in those infected with SARS-CoV-2 compared to the non-infected population, as demonstrated by this meta-analysis, should indicate that these cases are directly related to SARS-CoV-2, regardless of pathophysiology. Additionally, there is an increasing usage of the Dallas criteria to confirm myocarditis in the setting of both SARS-CoV-2-induced and COVID-19 vaccine-induced myocarditis [18]. Dallas criteria states that the diagnosis of myocarditis is dependent on the presence of “inflammatory infiltrate of the myocardium with necrosis and/or degeneration of adjacent myocytes, not typical of ischemic damage associated with coronary artery disease” [19]. Conversely, the currently accepted diagnostic criteria for Takotsubo cardiomyopathy states that there must be the absence of confirmed or suspicion of underlying myocarditis [20]. When taking these two sets of criteria into consideration, the gray area that has been suggested between SARS-CoV-2-induced myocarditis and Takotsubo cardiomyopathy is more black and white than suggested in the Haussner study.

Additionally, some studies have demonstrated a significantly increased risk of myocarditis in those who received the COVID-19 vaccination compared to that of the general population. This conclusion is also dependent on the demographics of the individuals. One study found a significantly increased risk of this sequelae in adolescents compared to adults and in male adolescents compared to their female counterparts [10]. However, current data suggest that this sequelae has an estimated rate of 52.4 to 105.9 cases per million doses administered. Additionally, most cases appeared to be self-limiting, with the majority of patients only requiring supportive treatment [21]. Therefore, while this complication exists, it is not only on a much smaller scale than that of SARS-CoV-2-induced myocarditis, but it is also much milder in symptomatology and prognosis. Additionally, it cannot be definitively concluded that either the result of the Won Lee study or the results of the randomized controlled trials can be generalized to the entire population, and even among male adolescents, there is now sufficient data to suggest that the vaccination carries rare sequelae of myocarditis and an even rarer risk of serious disease resulting from this sequelae.

Several limitations of this study include the sample size of the data set being analyzed in this meta-analysis, which is also a result of the relative novelty of the subject matter. A limitation specific to meta-analysis is that by combining results from multiple sources, there is an ever-present risk of skewing the data set. This can contribute to the overall validity of the study and the conclusions drawn. Additionally, at the height of the COVID-19 pandemic, there were many more sick patients, leading to skewed data. Therefore, all conclusions within this study must be considered in tandem with other available evidence and the original studies being analyzed. Moreover, all conclusions drawn from this study do not necessarily represent a definitive answer to the importance of COVID-19 vaccination, and therefore, no clinical decision should be made based on this preliminary data.

Future directions include the compilation of a more extensive data set to increase the statistical power of the meta-analysis. Additionally, COVID-19 has been associated with a variety of other cardiac and non-cardiac sequelae, and therefore, the future inclusion of these other conditions will allow for the better generalization of the protective effect of COVID-19 vaccination.

## Conclusions

Upon a meta-analysis of a compilation of previous studies examining the incidence and relative risk of SARS-CoV-2-induced myocarditis, there was a significantly higher average risk among those with an unspecified vaccination status or those who were unvaccinated compared to the non-infected population. Noting the insignificant difference in acquired myocarditis following infection with COVID-19 between those who are vaccinated, it can be concluded that infection with SARS-CoV-2 in the unvaccinated carries a significant risk of myocarditis and that the vaccination is protective of this sequelae. These results support the continued COVID-19 mRNA vaccination for all those medically able to receive it.
